# Potential vascular confound in GPR84-PET imaging following systemic LPS administration

**DOI:** 10.1073/pnas.2619004123

**Published:** 2026-09-28

**Authors:** Sami S. Zoghbi, Robert B. Innis

**Affiliations:** ^a^Molecular Imaging Branch, National Institute of Mental Health, NIH, Bethesda, MD 20892-1026

We read with interest the recent report by Kalita et al. describing [^18^F]MGX-110S as a GPR84-targeted Positron Emission Tomography (PET) tracer for imaging neuroinflammation (1). While the tracer development is compelling, systemic lipopolysaccharide (LPS) administration introduces vascular inflammatory effects that may confound interpretation of the PET signal as purely parenchymal microglial activation.

Systemic endotoxemia induces well-characterized peripheral immune responses, including activation of monocytes (2) and the coagulation cascade, leading to platelet–leukocyte–endothelial interactions at sites of vascular inflammation. Increased leukocyte adhesion to the endothelium during endotoxemia has been documented (3). In a canine endotoxemia model (4), scintigraphic studies demonstrated rapid and irreversible sequestration of ^111^In-labeled platelets and leukocytes within the pulmonary microvasculature, accompanied by profound leukopenia that persisted for hours (4). This leukopenia reflects margination driven by upregulated adhesion molecules (3). Although most evident in the lung, similar processes may occur in other capillary beds such as the cerebral capillaries (5).

Because GPR84 is highly expressed in circulating myeloid cells (6), LPS-induced leukocyte adhesion within cerebral capillaries may contribute to the observed PET signal. Radiotracer binding to adherent intravascular cells could potentially be misinterpreted as parenchymal uptake, particularly given the limited ability of standard perfusion methods to remove adherent leukocytes (7). Although systemic LPS exhibits limited direct penetration across the blood–brain barrier under typical endotoxemia conditions, peripheral inflammatory signaling can nevertheless induce central neuroimmune responses, including microglial activation. In addition, systemic LPS including intraperitoneal injections (8) has been reported to disrupt blood–brain barrier integrity (9, 10), potentially altering tracer delivery and facilitating entry or retention of peripheral immune cells at the neurovascular interface. Consequently, the PET signal may reflect contributions from vascular-associated myeloid cells, altered blood–brain barrier permeability, and genuine parenchymal neuroinflammatory responses, complicating attribution specifically to resident microglial activation.

Additional validation—such as intracerebral LPS models, vascular signal correction, or depletion of circulating myeloid cells—would help distinguish vascular from parenchymal contributions. These considerations do not diminish the promise of the tracer but suggest that the specificity of the PET signal for central microglial activation requires further confirmation.

## Data Availability

There are no data underlying this work.

## References

[1] M. Kalita , Illuminating proinflammatory myeloid cells with PET tracers targeting GPR84. Proc. Natl. Acad. Sci. U.S.A. **123**, e2536372123 (2026). 10.1073/pnas.2536372123 PMC13214000 42166242

[2] M. Guha, N. Mackman, LPS induction of gene expression in human monocytes. Cell. Signal **13**, 85–94 (2001). 10.1016/s0898-6568(00)00149-2 11257452

[3] B. Jilma , Regulation of adhesion molecules during human endotoxemia. No acute effects of aspirin. Am. J. Respir. Crit. Care Med. **159**, 857–863 (1999). 10.1164/ajrccm.159.3.9805087 10051263

[4] H. D. Sostman , Platelet kinetics and biodistribution in canine endotoxemia. Invest. Radiol. **18**, 425–435 (1983). 10.1097/00004424-198309000-00005 6358113

[5] H. E. de Vries , Lymphocyte adhesion to brain capillary endothelial cells in vitro. J. Neuroimmunol. **52**, 1–8 (1994). 10.1016/0165-5728(94)90155-4 7911476

[6] M. Suzuki , Medium-chain fatty acid-sensing receptor, GPR84, is a proinflammatory receptor. J. Biol. Chem. **288**, 10684–10691 (2013). 10.1074/jbc.M112.420042 PMC3624448 23449982

[7] H. Hu, J. Quintana, R. Weissleder, S. Parangi, M. Miller, Deciphering albumin-directed drug delivery by imaging. Adv. Drug Deliv. Rev. **185**, 114237 (2022). 10.1016/j.addr.2022.114237 PMC9117484 35364124

[8] W. A. Banks, S. M. Robinson, A. Verma, M. A. Nakaoke, Lipopolysaccharide-induced blood-brain barrier disruption: Roles of cyclooxygenase, oxidative stress, neuroinflammation, and elements of the neurovascular unit. J. Neuroinflammation **12**, 223 (2015). 10.1186/s12974-015-0434-1 PMC4660627 26608623

[9] S. B. Reddiar , A biodistribution study of the radiolabeled Kv1.3-blocking peptide DOTA-HsTX1[R14A] demonstrates brain uptake in a mouse model of neuroinflammation. Mol. Pharm. **20**, 255–266 (2023). 10.1021/acs.molpharmaceut.2c00614 36331024

[10] H. Zhang , Visualization of blood-brain barrier disruption in septic mice with the new method based on in vivo imaging technology. Neurocrit. Care **41**, 925–941 (2024). 10.1007/s12028-024-02018-x 38982003

